# Stigmatization of neurodivergence in the workplace

**DOI:** 10.3389/fpsyg.2026.1818600

**Published:** 2026-05-21

**Authors:** Chloe R. Cameron

**Affiliations:** Ivey Business School, Western University, London, ON, Canada

**Keywords:** concealable identity, disclosure, diversity, inclusion, invisible difference, neurodiversity, stigma

## Abstract

Neurodistinct (e.g., autistic, dyslexic, ADHD) people, who comprise 17–20% of the population, continue to be stigmatized in the workplace. Several bodies of literature address the stigmatization of neurodivergence, but a comprehensive model of how it arises in organizations does not exist, particularly as it relates to socio-contextual and perceiver-based factors. Some existing empirical accounts in which neurodistinct stigmatization is evident currently do not align with theoretical explanations, drawing attention to the need for more comprehensive conceptualization. Without such a model, theorizing about disclosure decisions and identity management, and organizational practice and policy adjustments to support neurodiversity, are incomplete. In this paper, I address this issue through a conceptual integration; I build on a stigma theory informed interaction model to trace pathways of potential neurodistinct stigmatization with a particular focus on characteristics of the social setting and the perceiver. I identify three bases of neurodistinct stigmatization—label, invalidation, and deviance—which each imply different perceiver experiences and interactional outcomes. I further discuss how the model contributes to understandings of identity management and organizational action to support workplace neurodiversity.

## Introduction

Stigmatization of neurodivergence (neurodevelopmental differences such as autism, dyslexia, and ADHD) in organizations is widely prevalent (Santuzzi and Keating, 2022) yet not well understood. Stigmatization reduces a target person in a perceiver’s mind “from a whole and usual person to a tainted, discounted one” (Goffman, 1963; p. 3). For the 17–20% of the population who are neurodistinct^1^ (Doyle, 2020; Cameron, 2021), this stigmatization results in workplace difficulties like higher rates of un- and underemployment, lower pay than others, and psychological strain from attempting to conceal differences (Santuzzi and Keating, 2022). Despite these negative impacts, the process of how and why neurodistinct people become stigmatized under different circumstances in the workplace remains relatively undertheorized.

Prior research has explored the behavioral differences of neurodistinct people that are associated with challenges in organizations (Doyle, 2020), and neurodistinct experiences of stigma (e.g., Johnson and Joshi, 2016; Nario-Redmond et al., 2019). Other theorists have extended disclosure and identity management theory to neurodivergence (Santuzzi and Keating, 2022), uncovered different expressions of stigmatization against people with disabilities (e.g., Hein and Ansari, 2022), documented organizational neuroinclusion efforts (Hennekam and Follmer, 2024), developed theories of neuroinclusion (Khan et al., 2022; Tomczak et al., 2025), and even created practitioner guides on “best practices” (e.g., Annabi et al., 2021). While work across these domains has undoubtedly contributed to a better understanding of the role of—and outcomes for—the neurodistinct person in the stigmatization process, and suggested ways that organizations can reduce potential stigmatization, it remains detrimentally siloed.

Theoretical integration can address two notable gaps: first, management-focused work often assumes that the reason for neurodistinct stigmatization is the label (e.g., “autism” or “dyslexia”) or does not specify the basis of stigmatization being discussed. To be sure, invisible disabilities disclosure studies show that workplace treatment varies depending on whether the “disability label” is made available (Santuzzi et al., 2014), implying the same is true for neurodivergence. As such, researchers have studied the content of stereotypes and understandings of individual forms of neurodivergence and invisible disabilities to support the development of strategies to overcome associated stigmatization (e.g., Turnock et al., 2022; Masuch et al., 2019). However, careful review of parallel empirical work shows that stigmatization is not necessarily due to named conditions (e.g., Romualdez et al., 2021; Nario-Redmond et al., 2019). How and when stigmatization is based on the label versus other factors—and what associated organizational implications are—remains unclear.

Second, thorough consideration of socio-contextual and perceiver-based factors in the neurodistinct stigmatization process is limited. Such factors may provide insight into the emergence of stigmatization that is not associated with perceptions of the neurodistinct label. For example, in their paper, Khan et al. (2022) theorize reverse discrimination as a majority group-level factor to manage to create neuroinclusive organizations. While their framework implies the existence of stigmatization based on social dynamics other than the neurodistinct label itself, it does not systematically explore how stigmatization of neurodivergence arises. Its goal is to help organizations manage perceptions of neurodivergence and neurodivergence-targeted organizational support that is offered to employees. Though admirable, organizations are unlikely to become neuroinclusive without an understanding that integrates how socio-contextual and perceiver-based factors contribute to stigmatization and associated interventions in the first place.

While disclosure theory has briefly acknowledged that perceiver characteristics and organizational diversity climate are likely to affect disclosure (Clair et al., 2005), consideration has been limited to how the potential discloser perceives them as they choose to reveal or conceal their neurodistinct identity. Again, the conceptualization occurs within a framework that assumes that the neurodistinct label is the reason for stigmatization: it is a choice between label-based stigmatization on one hand, and no stigmatization but consequences of concealing on the other. Theory about how perceiver characteristics and socio-contextual factors affect the range of potential interactional outcomes—the actual choices—that are weighed in identity management decisions is absent.

In this paper, I address these omissions by integrating work from multiple domains including neurodiversity, invisible differences, and taken-for-grantedness in a stigma theory informed framework to theorize the pathways of potential neurodistinct stigmatization. A social interactionist application of stigma theory (Goffman, 1963; Jones et al., 1984) is particularly helpful because it focuses on behavioral, perceptual, and interpretive sequences in interactions. This enables me to theorize how perceiver expectations in interactions with a neurodistinct person are likely to evolve as interactional processes unfold and lead to potential stigmatization. The perspective also allows space to consider characteristics of the perceiver and social setting.

I make three main contributions: first, I identify and explicate three separate bases of neurodistinct stigmatization in the workplace: label, invalidation, and deviance. This answers calls to theorize the nuances of neurodistinct identity management (e.g., Johnson and Joshi, 2014), by illustrating additional pathways and complexity in neurodistinct disclosure decisions. Second, I identify bases of stigmatization that have been undertheorized—and potentially have yet to be addressed—in organizational neuroinclusion practices and connect them to theoretical solutions. By doing so, I develop a framework of how neuroinclusion can be supported more comprehensively in the workplace. Third, I draw attention to the role of perceivers in potentially stigmatizing interactional processes. This enables me to theorize how neurodistinct stigmatization may be more likely to arise between certain people in organizations and invite a deeper focus on managing variation in stigmatization in organizational neuroinclusion strategies.

### Stigmatization of neurodivergence

“Neurodivergence” is a term attributed to people who do not fit the typical cognitive profile (Doyle, 2024). While its application varies—and is contested (Doyle, 2020)—I draw boundaries based on functional differences that cut across neurotypes for two reasons. First, within-neurotype heterogeneity is considerable and limits theorizing based on specific conditions (e.g., Hughes, 2021). Second, across-group homogeneity relating to spiky profiles (i.e., greater than average variation in function across cognitive domains; Doyle, 2020) and pressure to conform to “neurotypical” social norms (Szulc and Staniszewska, 2025; Longmire et al., 2025) provides opportunities for broader theoretical application than focusing on individual differences. Thus, I apply a definition of neurodivergence that includes multiple neurological differences provided meaningful organizational contributions are possible. In addition to the above commonalities, neurodistinct groups share the experience of stigmatization at work.

Similarly to institutional theory, stigma theory posits that taken-for-granted social norms define and enforce what is considered deviant. These norms inform interactional expectations, guide behavior, and shape interpersonal judgments. Stigmatization occurs when one interactant devalues another due to the perception that the other deviates from the prevailing arrangements and expectations (Goffman, 1963, 1967), either because of “flaws” or behaviors (Aranda et al., 2023). As Aranda et al. (2023) note, “attributes or labels are not stigmatizable *per se* but are stigmatized based on the perceptions and interactions between audiences in a given place/time” (p. 1341, italics in original). Stigmatization, then, is a dynamic, relational process involving interactions between the perceiver and potential target of stigmatization, situated in their social context. Unfortunately, management and organization theories have almost exclusively focused on the potential target and their management of stigma (Pollock et al., 2019; Zhang et al., 2021) and, as I will illustrate, organizational neurodiversity theories are likewise incomplete.

Five characteristics of stigma have been identified: concealability, controllability, centrality, disruptiveness, and malleability (Zhang et al., 2021). These affect the degree and type of stigmatization that is enacted. Studies of invisible differences such as neurodivergence generally focus on the dimensions of concealability and centrality, due to their relationship with and importance to disclosure decisions and identity management (discussed below). Other characteristics have been less of a focus in neurodivergence-related literature.

Stigmatization is experienced and expressed in multiple forms. While most work focuses on hostile expressions, there are also “benevolent” versions. Hostile forms are characterized by antipathy, while benevolent ones are underpinned by positive intentions on the part of the perceiver, often leading to prosocial behaviors (Glick and Fiske, 1996; e.g., Hein and Ansari, 2022). Nevertheless, both forms involve the perceiver devaluing the other person. While I mainly focus on the emergence of stigmatization rather than its expression, the critical insight from this work is that different expressions of stigmatization imply different affective experiences of stigmatization on the part of the perceiver. This consideration is only briefly raised in prominent theorizing (e.g., Zhang et al., 2021) but has significant implications for understanding stigmatization given its differential effects on subsequent perceiver behavior.

Insights about how and when stigmatization of neurodivergence occurs can be drawn from work on invisible differences, individual medically defined conditions, and organizational neurodiversity inclusion.

#### Stigmatization of neurodivergence as an invisible difference

Different forms of neurodivergence are often conceptualized as concealable stigmatized identities or invisible disabilities (Santuzzi and Keating, 2022). Much of the prior work relating to stigmatization in those domains focuses on disclosure and identity management. Applied to neurodivergence, disclosure is when a neurodistinct person reveals their neurodistinct identity to others.

Most disclosure theory focuses on the trade-offs that an individual must make between (1) the psychological strain and associated consequences to well-being that they face because they cannot share their “whole selves” in the workplace and (2) the label-based stigmatization that they experience when they do (Follmer et al., 2020). The psychological strain referenced arises from efforts to suppress innate tendencies that would expose the individual as being part of the neurodistinct group and is called “masking” (e.g., Radulski, 2022), “camouflaging” (e.g., Ai et al., 2022), or “passing” (e.g., Libsack et al., 2021).

The degree to which masking is effective for neurodistinct people is likely to vary within and across neurodistinct groups as well as context (e.g., Johnson and Joshi, 2014; Ragins, 2008), representing an underdeveloped area of neurodivergence disclosure theory. Ragins (2008) alludes to some variability in masking abilities, and Jones and King (2014) briefly discuss stigma visibility but elaborate only on stigmas with a specific “course” (e.g., pregnancy). An underlying assumption of most work is that individuals with permanent differences can conceal if they choose to, leading to calls for more specific theorizing on neurodistinct disclosure (e.g., Johnson and Joshi, 2014).

The type and severity of the social consequences (i.e., expression of stigmatization) depend on the specific identity (e.g., bisexual identities are more severely rejected than gay ones; Ragins, 2008), indicating that stigmatization due to a “tarnished” identity is a nuanced process that depends on the perceiver’s understandings and evaluations of the specific identity. Invisible disabilities, including some neurodistinct conditions, tend to have lower levels of social awareness, causing additional difficulties for workers with invisible differences compared to workers with visible conditions (Santuzzi et al., 2014); perceivers “[use] their (lack of) knowledge of various disabilities to determine whether the confederate’s condition … constituted a legitimate disability” (Santuzzi et al., 2014 citing Carpenter and Paetzold, 2013; p. 214).

Some attention has been paid to understanding these evaluation processes as it relates to granted accommodations in the disability literature, pointing to the importance of perceptions of procedural and distributive fairness (e.g., Colella, 2001; Colella et al., 2004). However, similar evaluation processes under different circumstances have not been studied—and implications for neurodistinct stigmatization processes are unclear.

While disclosure models theorize that certain relational factors (e.g., trust; Clair et al., 2005; Ragins, 2008) and perceiver characteristics (e.g., general disposition; Clair et al., 2005) affect disclosure decisions, it is within frameworks that center potential label-based stigmatization. The possibility of stigmatization without disclosure is also not typically addressed in this body of work.

#### Stigmatization of neurodivergence as individual conditions

Work has also been done to understand stigma associated with—and other social evaluations of—individual conditions that are categorized as neurodistinct. For example, Johnson and Joshi (2016) explored factors that affect the stigma experiences of autistic individuals in the workplace, concluding that age of diagnosis and whether an individual discloses at work both have important implications. A small study on public perceptions of autism showed that individuals uneducated on autism saw autistic people as being unable to understand social rules, incapable of independent living, and/or having a mental disability (Huws and Jones, 2010), which are all likely to be associated with stigmatization.

Turnock et al. (2022) theorize that autistic stigma is a function of (1) public and professional understanding of autistic traits and (2) the interpretation of visible autistic traits, moderated by macro-cultural factors, sex/gender, individual differences, and disclosure. Together, these studies indicate that autistic stigmatization is affected by characteristics of the social context, perceiver, and stigmatized person but studies continue to focus disproportionately on the stigmatized person or group, with other factors acting as more background moderators than central inputs.

Studies of stigmatization of other neurodistinct groups also generally focus on the stigmatized individual (e.g., Nalavany et al., 2015; Wissell et al., 2022). However, work on stereotypes associated with neurodistinct conditions lends insight into how various neurodistinct groups are perceived. For example, dyslexic people are stereotyped as “dumb, lazy, and incapable of learning to read and spell words” (Odegard and Dye, 2024; Washburn et al., 2011); ADHD stereotypes include a lack of attention, hyperactivity, impulsive behavior, academic underachievement, family problems, and undesirable risk-taking (Masuch et al., 2019). These negative group-level categorizations indicate that stigmatization of these identities undoubtedly occurs, and that the perceptions of the stigmatizer are central to this interpretive process.

In a study of perceptions of people with mental illnesses, findings showed that judgments on the dimensions of warmth and competence particularly affect perceptions. While all were stigmatized, the authors found that perceivers had vastly different attitudes towards individuals based on specific combinations of those dimensions (Sadler et al., 2012). How these categorizations—or other dimensions of affective significance—may map to perceptions of neurodistinct people in the workplace, though, has not been studied.

#### Stigmatization of neurodivergence addressed in organizational inclusion theory

Additional work has been done to document the types of organizational practice and policy changes intended to help support neurodivergence and theorize how these contribute to neuroinclusion (Hennekam and Follmer, 2024; Khan et al., 2022; Volpone et al., 2022; Doyle, 2020). All organizational adjustments are, presumably, meant to address some organizationally embedded expression of stigmatization. Since I am attending to the process of stigmatization emergence rather than its embedded structural expressions (e.g., job design, compensation policies), I focus on organizational changes that directly relate to interpersonal interactions. Such adjustments have been documented in selection practices (i.e., interview processes) and neurodiversity-related training.

Organizations have moved towards skills- and strengths-based assessments in the recruitment process to mitigate potential neurodistinct stigmatization and bias in traditional interview processes (Hennekam and Follmer, 2024; Ovaska-Few, 2018). This strategy acknowledges and circumvents the stigmatization that may arise in interview processes but does so by reducing dependence on social judgment rather than attempting to mitigate stigmatization within it. While this may be one strategy to reduce stigmatization, eliminating social judgments does not lend insight into the emergence of stigmatization and is unlikely to be a viable strategy in other workplace situations.

Conversely, training appears to be a mechanism that organizations use in attempt to change outcomes of interactions rather than eliminate reliance on them. Again, often efforts are focused on the neurodistinct person (e.g., “job-readiness” training). The implication is that exhibited neurodistinct behaviors may become stigmatized and the person could work to change them to align with accepted ways of behaving. This is a socialization strategy, aiming to adapt the individual to the organization rather than vice versa. While socialization is not *necessarily* negative, it can be—to the extent that recommended adaptation demands neurodistinct masking.

Managers and colleagues often also undergo training to better support neurodiversity. These trainings tend to focus on “raising awareness and acceptance of neurodiversity while also trying to minimize unconscious biases” (Hennekam and Follmer, 2024; p.7). Examples include how Ultranauts provides training to support equitable career development and more adaptive communication styles, and other corporate “neurodiversity awareness” training for employees and managers in hopes that they will subsequently better support neurodistinct (often autistic) colleagues (Hennekam and Follmer, 2024).

Volpone et al. (2022) suggest that manager and coworker training should cover common behaviors associated with neurodivergence, highlight similarities between the support needs of neurotypical and neurodistinct people, and feature real examples and neurodistinct voices. These examples and suggestions show where practitioners have observed interactional friction. Inferable issues include bias, communication incompatibilities, a lack of understanding of neurodivergence, perceptions based on stereotypical representations, and a lack of familiarity with neurodivergence among neurotypical workers. While these inferences do not explain the emergence of neurodistinct stigmatization, they imply interactional moments and factors that are potentially significant in the stigmatization process.

Khan et al. (2022) theorize that perceptions of reverse discrimination need to be actively managed to create neuroinclusive organizations. They do not expressly conceptualize this as a mechanism to reduce stigmatization characterized by high disruptiveness (i.e., threat; Zhang et al., 2021), however, they do indicate that perceptions of preferential treatment can threaten neurodiversity inclusion efforts. They theorize that negative perceptions arise when the dominant group perceives the same treatment is needed for all. Interestingly, they acknowledge that majority group members who are otherwise disadvantaged often have even more negative perceptions of diversity initiatives but do not further engage with potential variation in the stigmatization process and implications for organizational efforts.

Taken together, much work has been done to understand and address neurodistinct stigmatization in organizations. However, notable gaps relating to the perceiver and social context remain, leaving certain empirical accounts of stigmatization undertheorized. These must be closed to develop a comprehensive understanding of the phenomenon and develop strategies to overcome it.

### Pathways of (potential) neurodistinct stigmatization

To develop my conceptual model, I draw on interaction models proposed by Jones et al. (1984), which explicate the sequential processes in human interactions. The interaction sequencing focuses primarily on interactants’ expectations and how these evolve due to stimulus cues, perceptions, and attributions at different times throughout ongoing interactions. Following interactional expectations over time enables me to pinpoint how and when neurodistinct stigmatization may arise.

There are two ways in which I draw on literature to theorize neurodistinct stigmatization; the first is to theorize interactional processes in social contexts and the second is to theorize the content of applied meanings of neurodivergence. For the former, I apply social interaction theory (Goffman, 1967) to conceptualize and situate initial interactional expectations in the prevailing norm setting, work on taken-for-grantedness (Steele, 2021) to theorize how misalignments between expectations and observations are perceived and dealt with, and work on invisible differences to map potential interactional variations. For the latter, I draw on work relating to the cultural meanings (Becker and Arnold, 1986) and stereotypes (van Knippenberg and Dijksterhuis, 2000) associated with social groups. I further review work to identify perceiver-based factors that have been shown to affect how the stigmatization process unfolds either by changing how the content of social meanings of neurodivergence are applied or affecting how the interactional process unfolds.

I first map the pathways of stigmatization emergence by tracing interactional processes and factors that affect specific—but not all—pathways. Then, I discuss additional perceiver-based factors that affect the broader model.

#### Starting point: the perceiver’s normative expectations

Interactional expectations are rooted in the social context (Jones et al., 1984; Goffman, 1967). The social and cultural history of a given context informs the norms, values, and beliefs that guide interactional expectations and behaviors, and act as parameters to determine what is and is not acceptable (Becker and Arnold, 1986; Goffman, 1967). Depending on how a society evolved, one or more strategies—ranging from mild to severe—may be in place to promote certain behaviors and deter others, determining expectation content not only for interactions themselves but also the consequences of (dis)obeying these often-unspoken rules (Bicchieri, 2014). Social arrangements can vary on countless dimensions yet serve the same purpose across contexts: to maintain what is considered desirable social behavior.

**Figure 1:**
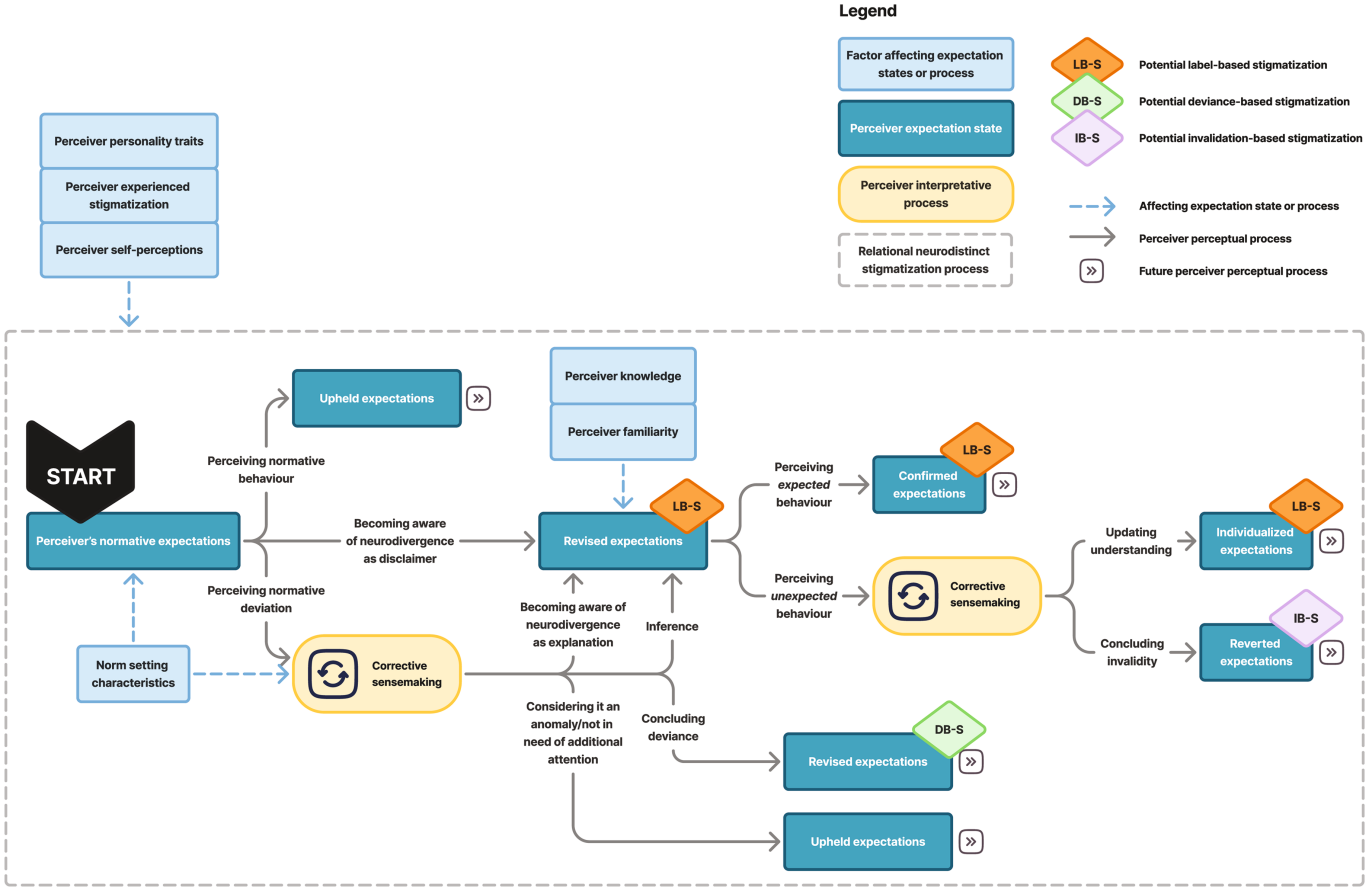
Pathways of (potential) neurodistinct stigmatization.

Perceivers’ individual expectations may vary depending on multicultural experiences, bringing certain ideals from their native cultures into other contexts (Oliveira and Stevanovic, 2025) and/or making them more tolerant of intergroup differences (Tadmor et al., 2018). The combination of these factors constitute the perceiver’s normative expectations, informing the starting point in my conceptual model (presented in Figure 1).

#### Pathways involving initial disclosure

When a perceiver is made aware of a neurodistinct identity early in the interaction sequence, it changes subsequent interaction expectations. Colella et al. (2004) theorize that the nature of the specific difference is among the most critical determinants of organizational views and treatment of people with disabilities. However, an important amendment is needed to theorize the emergent stigmatization process: it is not the nature of the neurodistinct identity or its specific expression that is most critical, but rather the *perceiver’s understanding* of the nature of the disclosed neurodistinct label—and associated expectations. This understanding is informed by the perceiver’s knowledge and familiarity with the neurodistinct identity in question.

##### Knowledge

For many, cultural meanings and stereotypes constitute the extent of knowledge content and form the cognitive basis of interpretations of people with invisible differences (Fox et al., 2018; Becker and Arnold, 1986). Stereotypes are mental representations of social groups (van Knippenberg and Dijksterhuis, 2000; Stangor and Schaller, 2000), “[containing] knowledge of what the group or members of the group in question are like” (Van Knippenberg and Dijksterhuis, 2011; p. 107). People use such categorizations to quickly interpret everyday social situations and have positive as well as negative implications (Bodenhausen et al., 2012).

Local cultural and historical events form socio-contextual perceptions, constituting the basis of categorical knowledge (Becker and Arnold, 1986). For example, Doyle (2020) overviews some of the historical events (e.g., the industrial revolution, the rise of literacy) that led various forms of neurodivergence to become salient social differences, illustrating that these categorizations emerge based on prevailing societal arrangements rather than objectively meaningful social boundaries.

However, knowledge can go far beyond prevailing perceptions and stereotypes both in depth and framing. For example, understandings that are based on medical literature (which problematizes and seeks treatments/cures at the individual level) will intuitively lead to different perceptions than knowledge based on the neurodiversity paradigm (which problematizes and seeks adjustments to prevailing social arrangements), multidisciplinary understandings, or no knowledge beyond social stereotypes.

Specific aspects of knowledge are likely to be particularly relevant in determining whether and how neurodistinct stigmatization occurs. For example, as mentioned above, studies of stereotypes of mental disorders indicate that competence and warmth play a particularly important role in determining perceiver attitudes (Sadler et al., 2012). This can be explained using Zhang et al.’s (2021) framework outlining stigma characteristics; perceived hostility (low perceived warmth) indicates high perceived disruptiveness (threat or danger), while high perceived warmth but low perceived competence is likely to be interpreted not as threatening but instead as a flaw that is uncontrollable. These characteristics are linked to different affective responses (here, fear versus pity). Thus, a perceiver’s cognitive understanding of a neurodivergence affects their expectations of neurodistinct behavior, their own affective state, and determines whether stigmatization occurs.

Importantly, even accurate knowledge about a social group without exposure to individuals from that group *reduces* individualization and increases the application of stereotypes (Smith et al., 2006), likely because it can lead perceivers to believe that they know more about a neurodistinct individual than they do.

##### Familiarity

Familiarity, on the other hand, represents the depth and breadth of personal experience with the particular neurodistinct group and individual. Categorically, depth of experience refers to how intimately a person has interacted with people with the specific difference. For example, work experience will differ from familial experience (Corrigan and Nieweglowski, 2019). Breadth of experience refers to the variety of people with the difference with whom a person has interacted. For example, exposure could be limited to a single person or several people of different ages and backgrounds who share the difference. While there is limited work on how breadth of experience with people with a given form of neurodivergence affects subsequent stigmatization independently of depth of experience, there is evidence that broad exposure to visible minorities leads to decreases in negative attitudes over time (Pettigrew and Tropp, 2006, 2008; Pauker et al., 2018), which could indicate that the same is true for other differences.

The relationship between familiarity and stigmatization is complex and non-linear. A review of studies on the stigmatization of individuals with mental conditions reveals a U-shaped relationship, with moderate familiarity (e.g., that of friends or coworkers of people with mental conditions) associated with the lowest levels of stigmatization and low familiarity (e.g., that of people with no experience or acquaintances) and high familiarity (e.g., that of service providers or family members) associated with higher levels (Corrigan and Nieweglowski, 2019). The initial reduction may be because first-person experience with individuals from a group enables a target to appreciate the individualism and value of each person rather than applying group-level expectations to individuals. However, factors such as family burden and associative stigmatization may lead to increased stigmatization (Solomon and Draine, 1995).

Other work indicates that perceiver familiarity with the individual fundamentally changes how categorizations are applied. Specifically, it reduces the automaticity with which categories are applied, instead prompting more conscious reflection in the social construal process (Quinn et al., 2009).

##### Post-disclosure interactions

Together, perceiver knowledge and familiarity constitute the perceiver’s existing understanding of the disclosed neurodistinct label and, thus, inform post-disclosure expectations. It is possible, based on this existing understanding, that the neurodistinct person becomes stigmatized immediately following disclosure. I call this *label-based stigmatization*.

If subsequently observed behavior aligns with these revised expectations, then the perceiver has their expectations confirmed and maintains whatever degree of label-based stigmatization was associated with their understanding. However, an alternative pathway of potential stigmatization is possible even if initial disclosure does not lead to stigmatization. Research on disclosure experiences indicates that the perceiver does not necessarily accept the disclosed identity as being true. Santuzzi et al. (2019) found that invalidation (defined as “skepticism about the legitimacy of the disability claim”) was one of the top concerns of employees with invisible disabilities in the workplace as they navigated identity management decisions. Invalidation might arise if the perceiver’s revised expectations for the neurodistinct person were not met (i.e., they observed behavior that was different from their revised expectations).

Drawing on theory on taken-for-grantedness, when observed behaviors do not align with expectations, people engage in a corrective sensemaking process—they search for plausible explanations to realign the perceived behaviors with their understandings (Steele, 2021). There are two possible outcomes: either the perceiver determines that their previous understanding of the neurodivergence was incomplete and updates it accordingly (i.e., learns) or the perceiver determines that the neurodistinct label is invalid.

This process is further complicated in organizational settings because disclosure can imply the receipt of accommodations. Thus, a disclosed label could be invalidated in two ways: either the perceiver does not believe that the disclosing individual is affected by the disclosed difference at all, *or* the perceiver believes that the discloser is exaggerating the extent of their difference to get an unfair advantage. Either way, the disclosing person is perceived as being untruthful—a threat.

Colella (2001) theorized that judgments about how much an individual needs granted accommodations affect whether coworkers perceive accommodations as being fair—and perceived fairness determines the attitudes of coworkers towards those receiving accommodations. This supports the argument that perceiving audiences engage in an evaluative process to determine both the motivation of disclosure (e.g., access to accommodations) and the validity and/or severity of the difference being disclosed. The perceiver’s understanding of neurodivergence and potential accommodations will directly affect the outcome of this corrective sensemaking process.

If the perceiver concludes that the stated neurodivergence is invalid, they are likely to revert to prior expectations, feel threatened, and stigmatize the neurodistinct person. I call this *invalidation-based stigmatization.* If, on the other hand, they are open to the possibility that their prior expectations were inaccurate or incomplete, they may update their understanding based on the individual’s expression of neurodivergence. This may still lead to label-based stigmatization, depending on how their learnings are integrated with prior understandings.

#### Pathways not including initial disclosure

If a perceiver is not disclosed to, interaction expectations are maintained, and they (at least initially) are unaware of any neurodivergence. Potential stigmatization can be avoided altogether if a neurodistinct person fully masks or is never in an interaction with the perceiver where neurodivergence is perceptible. For example, a dyslexic person may interact only verbally with a colleague in another department.

However, in non-disclosure scenarios, when a neurodistinct person’s behavior then is perceived to deviate from the perceiver’s normative expectations (via either a flaw in masking or lack of masking), it is likely to be perceived as a departure from normative, taken-for-granted ways of being. It has long been understood that ways of being become socially entrenched and taken for granted over time (Berger and Luckman, 1967) and that violations can lead to *deviance-based stigmatization* (Goffman, 1963).

Again, a corrective sensemaking process follows such perceived deviations (Steele, 2021). When it is normative expectations that are violated, Steele theorizes that the process is affected by four “choreographical characteristics.” These are choreographical fluidity, ordinary accountability, directorial sensegiving, and material scene-setting. Respectively, these characteristics describe the extent to which a norm context has clearly defined and narrow mutually accepted categorizations, how strongly individuals feel they must uphold normative ways of being, how strongly authority figures enforce upholding normative ways of being, and to what extent the physical context limits deviations from occurring. Together, these characteristics affect the likely incidence of deviations, and perceptions and interpretations of deviations that may lead to deviance-based stigmatization. Similar concepts describing normative tolerance levels for norm-deviating behavior have emerged in studies of national cultures (termed tight and loose cultures; Gelfand et al., 2011), and political science (aptly named the general tolerance norm; Lawrence, 1976).

Neurodistinct people are likely to benefit from contextual factors that make it more difficult to violate norms (e.g., environments with clear signage on intended usages) and that make it more likely that the perceiver will simply tolerate the behavior rather than enacting social consequences (higher general tolerance/looser cultures). In this sense, contextual factors affect the threshold at which stigmatizing judgments are made on perceived neurodistinct norm-violators.

There are three ways that this corrective sensemaking process can proceed. First, depending on the perceiver’s understanding of the particular form of neurodivergence—as discussed above—and other social categorizations that are associated with norm-deviating behaviors, they may *infer* an identity. If the perceiver correctly infers the neurodistinct identity, they may stigmatize the neurodistinct person based on this inferred difference, in alignment with the label-based stigmatization process. However, there may also be increased stigmatization if the identity is accurately inferred. As Elliott et al. (1982) theorize, “[i]t is assumed that people will make a claim of legitimacy only if they satisfy [the associated criteria]… Essentially, this prior norm states that the illegitimate actors should know their place and not try to be something they are not,” (p. 279). This meta-norm is perceived to be violated if a difference is inferred rather than disclosed.

If the perceiver incorrectly infers a different neurodistinct label or other social categorization that they believe explains the perceived behavior, the neurodistinct person may be subject to stigmatization based on a completely different label. The degree of stigmatization in this case is unpredictable because it is unclear which identity the perceiver incorrectly infers.

Second, the neurodistinct person may disclose their neurodivergence to *explain* the behavior. Upon receiving this explanatory disclosure claim, the perceiver undergoes a perceptual process like that described above in the disclosure scenario: they compare the perceived behavior with what they would expect given their knowledge of and familiarity with the neurodivergence and decide whether the two are in alignment. If they are in alignment, label-based stigmatization is a possibility, depending on the perceiver’s understanding of the neurodistinct label. Again, there may be a penalty over initial disclosure because disclaimers in social interactions are interpreted differently than explanations (Hewitt and Stokes, 1975) and because “relevant” qualifying information was initially withheld (Elliott et al., 1982).

If, on the other hand, the perceiver’s understanding of what they might expect from a person with the stated invisible difference does not align with perceived behaviors, invalidation-based stigmatization can, again, occur.

Finally, there may be no inference and no explanatory disclosure. In this case, stigmatization may occur depending on the above choreographical characteristics and other perceiver characteristics, discussed next.

All the pathways theorized here are part of potentially ongoing interactional sequences and therefore are not exclusive or terminal. As interactions continue, new forms of stigmatization can occur, existing forms of stigmatization can fluctuate, or altogether be eliminated through continued cycles of such interactional processes.

#### Perceiver characteristics

I further theorize that there are perceiver characteristics—personality, own level of experienced stigmatization, and self-perception— that affect the entire neurodivergence stigmatization model.

##### Personality traits

Openness to experience and agreeableness have been associated with different tendencies in potential stigmatization processes. For example, in a study of attitudes towards people with mental health issues, perceivers who were more open to experiences, and those who were more agreeable, were less likely to stigmatize (Steiger et al., 2022). While neurodivergence and mental health issues are not the same, they are both not immediately visible and both have to do with neurological differences that are often poorly understood and stigmatized in the general public (Parcesepe and Cabassa, 2013; Huws and Jones, 2010). These traits also are also intuitively applicable to the neurodistinct stigmatization process;

Agreeableness is likely to be particularly influential in the potential pathway to deviance-based stigmatization because those higher in agreeableness are inherently more concerned with the welfare of others and are less concerned about asserting power, which reduces the likelihood that they would arrive at a conclusion that devalues an interactant based on normative deviations (Roccas et al., 2002).

Openness to experience is likely to play a more significant role in the potential pathways to label-based or invalidation-based stigmatization: label-based stigmatization because those higher in openness to experience have more “understanding and tolerance for all people” (Parcesepe and Cabassa, 2013; p. 792), making it more likely that they will be open to iterating their interactional expectations through personal experience. Similarly, I theorize that higher openness to experience is likely to affect the invalidation-based pathway because the perceiver is more likely to be inquisitive about a discrepancy between prior expectations and observed behaviors, rather than quickly conclude that a disclosed difference is invalid.

##### Experienced stigmatization

Neurodistinct stigmatization is likely to be affected by the extent to which the perceiver has experienced stigmatization themselves. This further depends on perceived similarities, prevailing social norms, and the salience of personal stigmatization experiences.

Perceived similarity between stigmatized identities plays a pivotal role in intergroup attitudes. Craig and Richeson (2016) suggest that individuals who perceive their group’s experiences of discrimination as similar to those of other marginalized groups are more likely to exhibit solidarity and less likely to harbor negative attitudes. However, when stigmatized identities are perceived as dissimilar—such as race and neurodivergence—it can reduce the likelihood of solidarity. This can lead perceivers to distance themselves from other stigmatized groups (i.e., stigmatize them to a greater degree).

Perceived similarity within the neurodistinct group may also play a role. A phenomenon called “lateral ableism” refers to discriminatory attitudes and behaviors that occur within certain marginalized communities. It has been observed within autistic communities, where individuals—consciously or unconsciously—uphold neurotypical standards and hierarchies (Gurba et al., 2024). For instance, some autistic individuals distance themselves from others with more pronounced support needs or other stigmatized identities (e.g., race), reinforcing a hierarchy that values certain expressions of autism over others (Giwa Onaiwu, 2020). This internal stratification can lead to increased stigmatization.

Majority group norms also significantly influence how stigmatized individuals perceive and interact with other marginalized groups. Shapiro and Neuberg (2008) found that when stigmatized individuals, such as Black men, believe that the majority group endorses prejudice against another minority group, they may conform to these perceived norms by expressing prejudice themselves, especially in public. This behavior is driven by a desire to align with majority norms and avoid further marginalization, illustrating how perceived social expectations can perpetuate intergroup bias even among stigmatized people. In the context of neurodiversity, if prejudice against neurodistinct individuals is perceived as normative, other stigmatized individuals may conform to these norms, potentially increasing stigmatization.

Finally, the salience of one’s own stigmatized identity can enhance empathy. Galanis and Jones (1986) demonstrated that individuals who are reminded of their own experiences with stigmatization are more likely to show tolerance and understanding toward other stigmatized individuals. This finding suggests that increasing awareness of one’s own stigmatized experiences can foster empathy and reduce prejudice toward others facing different forms of stigmatization, including neurodistinct individuals. However, this salience is only likely to be favorable when perceptions of reverse discrimination are absent. Prior work shows that majority group members who are disadvantaged for other reasons are more likely to view negatively programs aimed at reducing other forms of workplace stigmatization (Isom Scott, 2018).

##### Self-perception

Self-perceptions of the perceiver may also affect the stigmatization process to the extent that they inform the base of stigmatization. For example, when a perceiver views themselves as being well-informed about a form of neurodivergence, they are likely to be more closed-minded if they perceive deviations from their expectations and be less likely to update their understandings (Ottati et al., 2015). This could result in higher incidences of invalidation-based stigmatization. While in-depth examination of how other self-perceptions may affect perceptions is limited, some can be intuited. For example, someone who perceives themselves as being inclusive may be less likely to engage in invalidation-based stigmatization but may be more likely to engage in label-based stigmatization that results in benevolent marginalization. To illustrate, a perceiver may believe that a neurodistinct colleague needs help communicating and restate or defend the person’s perspectives in team meetings when the neurodistinct person actually feels belittled by such behavior. Thus, self-perceptions may change the base and expression of stigmatization but may not operate the way that perceivers expect.

## Discussion

Stigmatization of neurodistinct people in the workplace is widespread, to the detriment of both neurodistinct individuals and organizations. While the phenomenon has been investigated in multiple domains, organizational research has not adequately explained the process of neurodistinct stigmatization emergence. It has instead primarily focused on the role of the neurodistinct person and how they can manage their “stigmatized” identity, and how organizations can change structural, uniform expressions of stigma. Here, I conceptualize neurodistinct stigmatization as an emergent process, specifically focusing on the role of the perceiver and the social context as interactional sequences unfold and expectations shift.

I provide a means to identify how and when neurodistinct stigmatization arises under different circumstances. By following the evolution of perceiver expectations through various interactional processes, I show that there are three different bases of neurodistinct stigmatization: label, invalidation, and deviance. Each of these has different implications both for interactants and organizations.

Table 1 summarizes generalizable profiles for each base of stigmatization, first providing exemplary descriptions from existing empirical work that imply each base, then highlighting notable characteristics, and, finally, describing implied practical interventions.

**Table 1:** Overview of bases of stigmatization and implied organizational interventions.

	Label	Invalidation	Deviance
Example description of empirical experience	“*It would make me think that they would not fit into normal society; that they would not be able to cope with living independently… That you’d live in, with your parents, your siblings, or maybe in you know a home, or something.”* (Huws and Jones, 2010; p. 338)	*“For those with more concealable disabilities, ableism was more likely to manifest as discounting the legitimacy of their embodied experiences in addition to invalidating their status as disabled, which felt dehumanizing.”* (Nario-Redmond et al., 2019: p. 745)	*“Not disclosing is not an option for me. If I do not disclose, I will either be forced to quit or be fired.”* (Romualdez et al., 2021, participant quote: p. 160)
Perceived issue	Inherent flaw	Unfair advantage/dishonesty	Not adhering to societal motivational schemes
Perceiver affect	Pity (depending on understanding)	Threat	Threat
Driving characteristic (Zhang et al., 2021)	Controllability/Variable (depending on understanding)	Disruptiveness	Disruptiveness
Primary factor(s) driving perception	Perceiver understanding of neurodivergence	Perceiver understanding of neurodivergence versus perceiver understanding of organizational inclusion practices	Characteristics of norm setting
Implied practical intervention(s)	Awareness training; exposure to people with specific neurodivergence	Awareness training; exposure to people with specific neurodivergence; trust-building and procedural fairness in organiza-tional support practices; reverse discrimination perception management	Establishing norms of inquisitiveness and willingness to revise expectations

Label-based stigmatization has been studied most commonly. Based on this model, prevalent interventions (e.g., awareness training; Hennekam and Follmer, 2024; Volpone et al., 2022) are likely to be effective. There are, however, two notable insights to be gleaned for the management of label-based stigmatization. First, while prior work has noted the importance of real-life examples and neurodistinct voices in training content (Patton, 2019; Volpone et al., 2022) and ensuring interactions between neurodistinct and neurotypical are meaningful (Khan et al., 2022), the importance of familiarity in building perceiver expectations has been underemphasized. As Smith et al. (2006) showed, knowledge without familiarity can lead to increased stereotyping, which indicates that awareness training that does not include exposure to neurodistinct people may, in some cases, be counterproductive. Second, given that different perceiver understandings can lead to different characteristics of stigmatization and perceiver affective states, it is important for organizations to consider the content of stereotypes associated with individual forms of neurodivergence.

Invalidation-based stigmatization has been considered to a lesser extent in the context of neurodivergence. Khan et al. (2022) discuss that management of reverse discrimination is necessary to create neuroinclusive workplaces. Elaborating on this insight, overcoming invalidation-based stigmatization involves managing both the interaction between perceiver understandings and organizational neurodiversity practices (aligning with Khan et al., 2022), as well as establishing trust that the organization engages in fair procedures to distribute support among all its employees (Colella et al., 2004). Reducing invalidation-based stigmatization involves not only awareness training, exposure to neurodivergence, and potentially necessary accommodations for neurodivergence, but also ensuring that employees from other groups are well supported in *their* work, unrelated to neurodiversity.

Deviance-based stigmatization has generally been backgrounded in neurodiversity work even though it underpins the entire phenomenon of neurodistinct stigmatization. It is only because of divergence *from something* that neuro*divergence* is a point of discussion. Yet this *something* only rarely enters the organizational neurodiversity conversation. As Longmire et al. (2025) note, “[b]ecause neurotypicality is the default in most organizations, it is difficult for non-autistic members of a workgroup or organization to see it as merely one pattern of interrelating” (p. 71). Doyle (2020) describes macro socio-contextual changes that brought different forms of neurodivergence into societal focus: “Dyslexia is discovered around the same time as literacy becomes mainstream through education; ADHD becomes more prevalent with the increasing sedentary lifestyles from the industrial revolution; autism increases in line with modern frequency of social communication and sensory stimulation and DCD as our day-today need for motor control of complex tools and machinery becomes embedded” (p. 112). But what about organizations today?

The model developed here indicates—using taken-for-grantedness theory (notably Steele, 2021)—how the organizational norm setting itself contributes to both the incidence of perceptions of deviance and perceivers’ compulsion to address them. This is not to suggest that social systems in organizations can be easily overhauled to accommodate neurodivergence. However, I argue that there are two important insights to be noted. First, explicating this pathway to neurodistinct stigmatization enables consideration of how far organizations are away from neuroinclusion. For example, if organizational norms are tight and highly enforced, more effort will be required to become neuroinclusive. Using this insight, organizations can design strategies that will be better suited to their current state. For instance, an organization that has a relatively loose norm setting may benefit more from an organizational focus to further embrace difference without disclosure, whereas an organization that has a tight norm setting may find that their resources are better spent on addressing label- and invalidation-based forms of stigmatization.

Second, consideration of deviance-based stigmatization opens conversations about which norms may be most conducive to neurodivergence. For example, by integrating insights from the perceiver characteristics analysis, organizations could foster norms of inquisitiveness and willingness to revise expectations to become a neuroinclusive organization where disclosure is not always necessary to be accepted and/or understood.

### Weighing all options

Neurodivergence disclosure theory can also be meaningfully advanced using this framework. To properly theorize disclosure and identity management, an understanding of which options are being weighed is critical. Prior work tends to imply that disclosure involves a choice between (1) potentially experiencing label-based stigmatization, and (2) concealing behavioral differences and facing associated intrapersonal consequences (Jones and King, 2014; Ragins, 2008). However, as I illustrate here, the decision in the case of neurodivergence is more complex.

My model shows that disclosure decisions for neurodistinct people are not simple “cost–benefit” analyses (Clair et al., 2005) comparing Options 1 and 2. Even as more recent work has highlighted the variability of disclosure decisions based on, for example, context (Ragins, 2008) and anticipated acceptance (Jones and King, 2014), and has begun to engage with neurodivergence specifically (Santuzzi and Keating, 2022), this paper theorizes additional complexity in the underlying disclosure framework, indicating additional bases of potential stigmatization on both sides of the disclosure decision. Neurodivergence can be the target of at least three different versions of stigmatization, which must all be carefully considered.

As Table 1 indicates, these pathways are each associated with different stigmatization characteristics (Zhang et al., 2021). Different configurations of stigmatization characteristics are likely to be associated with different perceiver emotions and, therefore, enacted differently. For example, a person may believe that autism is inherently detrimental and warrants pity (primary stigmatization characteristic: low controllability). If an autistic colleague then discloses to them—and the person judges the disclosure to be valid—the perceiver is likely to feel pity and enact benevolent forms of marginalization (e.g., Hein and Ansari, 2023). However, if the same autistic colleague discloses and then the person judges the disclosure as being invalid, they are likely to believe that the colleague is seeking an unfair advantage (primary stigmatization characteristic: disruptiveness), experience threat, and engage in a hostile expression. In short, different bases of stigmatization lead to different stigma characteristics, and different characteristics are associated with different perceiver affective states and, therefore, enactments of stigmatization.

Understanding the connections between perceiver affect, stigmatization characteristics, and associated expressions is critical; it changes the question of disclosure from, “Do I want to be potentially stigmatized or hide who I really am?” to, for example, “Do I want to be seen as a charity case or possibly a cheater—or hide who I really am but possibly be seen as strange?” It is not only a decision about whether to disclose but also a decision about which form of potential stigmatization are preferable. How neurodistinct people perceive and evaluate these options should be studied empirically to develop a better understanding of the nuances of neurodistinct disclosure.

### Bringing the perceiver to the foreground

Consideration of perceivers has been limited in organizational neurodiversity theory, yet it aligns conceptually with the social model of neurodiversity (Chapman, 2020), which problematizes social arrangements rather than neurodistinct behavior. Disclosure theory has noted the potential impact of relational characteristics (e.g., trust between the perceiver and potential discloser; Clair et al., 2005; Ragins, 2008) and briefly touched on perceiver disposition and demographics. For example, Clair et al. (2005) theorized that some people are good at “eliciting intimate information” (p. 86) and others may know more about and be sympathetic towards the difference. These are accurate insights but the mechanisms driving them were undertheorized. Here, I propose that it is personality traits that are likely to make some people more favorable targets for sharing personal information, and personal stigmatization experiences or familiarity that would cause them to be more sympathetic.

Disclosure and identity management theories ultimately focus on the decision of the person who will be potentially stigmatized. However, understanding perceiver characteristics that are associated with higher and lower levels of stigmatization under different circumstances is helpful because neurodistinct people are likely to be at least somewhat aware of them. Identifiable, categorical perceiver characteristics that are more likely to lead to positive outcomes are likely to manifest as perceived disclosure target profiles that neurodistinct people use to make identity management decisions.

Finally, consideration of perceiver characteristics associated with higher or lower levels of neurodistinct stigmatization is useful in design and implementation of organizational action to support neurodivergence. Elaborating on the recommendations of Khan et al. (2022) and Volpone et al. (2022), for example, potential managers can be matched by conducting personality assessments, trainings can incorporate exercises in which coworkers’ or managers’ own stigmatization experiences are brought to their attention, and education can be designed to include both knowledge and familiarity elements. Additional empirical work to test these theories would be greatly beneficial.

## Conclusion

Stigmatization of neurodivergence remains prevalent in organizations. Previous work has made in-roads to understanding associated social dynamics and organizational efforts to better support neurodistinct workers. In this paper, I overcome theoretical siloes to develop a situated model of neurodistinct stigmatization emergence in the workplace. I identified three bases of neurodistinct stigmatization, which have different organizational implications. I further discuss how work on organizational neurodiversity inclusion and identity management theories can be elaborated to inform organizations working to support neurodistinct workers, abilities, and insights.

## Footnotes

1 While “neurodivergent” is the more commonly used term, I use “neurodistinct” because it—to a slightly lesser extent—implies deviation from an acceptable norm. I acknowledge and accept that preferences vary.

## References

[ref1] Ai W. Cunningham W. A. Lai M.-C. (2022). Reconsidering autistic ‘camouflaging’ as transactional impression management. Trends Cogn. Sci. 26, 631–645. doi: 10.1016/j.tics.2022.05.002, 35641372

[ref2] Annabi H. Crooks E. W. Barnett N. Guadagno J. Mahoney J. R. Michelle J. . (2021). Autism @ Work Playbook: Finding talent and Creating Meaningful Employment Opportunities for People with Autism. 2nd Edn Seattle: ACCESS-IT, The Information School, University of Washington.

[ref3] Aranda A. M. Helms W. S. Patterson K. D. W. Roulet T. J. Hudson B. A. (2023). Standing on the shoulders of Goffman: advancing a relational research agenda on stigma. Bus. Soc. 62, 1339–1377. doi: 10.1177/00076503221148441

[ref4] Becker G. Arnold R. (1986). “Stigma as a social and cultural construct,” in The Dilemma of Difference, eds. Ainlay S. C. Becker G. Coleman L. M. (Boston: Springer), 39–57.

[ref5] Berger P. L. Luckman T. (1967). The social Construction of Reality: A Treatise in the Sociology of Knowledge. Springer: Doubleday.

[ref6] Bicchieri C. (2014). “Norms, Conventions, and the Power of Expectations,” in Philosophy of Social Science: A New Introduction, (Oxford, England: Oxford University Press).

[ref7] Bodenhausen G. Kang S. K. Peery D. (2012). “Social categorization and the perception of social groups,” in The Sage Handbook of Social Cognition, Susan T. Fiske, C. Neil Macrae (Eds.), 311–329.

[ref8] Cameron C. R. (2021). Calculating the incidence of neurodiversity in the general population. Available online at: https://www.ivey.uwo.ca/media/2hsbjv41/calculating-the-incidence-of-neurodiversity-in-the-general-population-v1-0822.pdf (Accessed October 15, 2025).

[ref9] Carpenter N. C. Paetzold R. L. (2013). An examination of factors influencing responses to requests for disability accommodations. Rehabil. Psychol. 58, 18–27. doi: 10.1037/a0030853, 23339323

[ref10] Chapman R. (2020) in Neurodiversity Studies: A New Critical Paradigm, eds. Rosqvist H. B. Chown N. Stenning A.. 1st ed (New York, NY: Routledge).

[ref11] Clair J. A. Beatty J. E. Maclean T. L. (2005). Out of sight but not out of mind: managing invisible social identities in the workplace. Acad. Manage. Rev. 30, 78–95. doi: 10.5465/amr.2005.15281431

[ref12] Colella A. (2001). Coworker distributive fairness judgments of the workplace accommodation of employees with disabilities. Acad. Manag. Rev. 26, 100–116. doi: 10.2307/259397

[ref13] Colella A. Paetzold R. Belliveau M. A. (2004). Factors affecting coworkers’ procedural justice inferences of the workplace accommodations of employees with disabilities. Pers. Psychol. 57, 1–23. doi: 10.1111/j.1744-6570.2004.tb02482.x

[ref14] Corrigan P. W. Nieweglowski K. (2019). How does familiarity impact the stigma of mental illness? Clin. Psychol. Rev. 70, 40–50. doi: 10.1016/j.cpr.2019.02.001 30908990

[ref15] Craig M. A. Richeson J. A. (2016). Stigma-based solidarity: understanding the psychological foundations of conflict and coalition among members of different stigmatized groups. Curr. Dir. Psychol. Sci. 25, 21–27. doi: 10.1177/0963721415611252

[ref16] Doyle N. (2020). Neurodiversity at work: a biopsychosocial model and the impact on working adults. Br. Med. Bull. 135, 108–125. doi: 10.1093/bmb/ldaa021, 32996572 PMC7732033

[ref17] Doyle N. (2024). “Defining neurodiversity and identifying neurominorities,” in Neurodiversity and Work, eds. Patton E. Santuzzi A. M. (Cham: Springer Nature), 13–38.

[ref18] Elliott G. Ziegler H. Scott D. R. (1982). Understanding stigma: dimensions of deviance and coping. Deviant Behav. 3, 275–300. doi: 10.1080/01639625.1982.9967590

[ref19] Follmer K. B. Sabat I. E. Siuta R. L. (2020). Disclosure of stigmatized identities at work: an interdisciplinary review and agenda for future research. J. Organ. Behav. 41, 169–184. doi: 10.1002/job.2402

[ref20] Fox A. B. Earnshaw V. A. Taverna E. C. Vogt D. (2018). Conceptualizing and measuring mental illness stigma: the mental illness stigma framework and critical review of measures. Stigma Health 3, 348–376. doi: 10.1037/sah0000104, 30505939 PMC6261312

[ref21] Galanis C. M. B. Jones E. E. (1986). When stigma confronts stigma: some conditions enhancing a victim’s tolerance of other victims. Personal. Soc. Psychol. Bull. 12, 169–177. doi: 10.1177/0146167286122003

[ref22] Gelfand M. J. Raver J. L. Nishii L. Leslie L. M. Lun J. Lim B. C. . (2011). Differences between tight and loose cultures: a 33-nation study. Science 332, 1100–1104. doi: 10.1126/science.1197754, 21617077

[ref23] Giwa Onaiwu M. (2020). I, too, sing neurodiversity. Ought J. Autistic Cult. 2, 58–67. doi: 10.9707/2833-1508.1048

[ref24] Glick P. Fiske S. T. (1996). The ambivalent sexism inventory: differentiating hostile and benevolent sexism. J. Pers. Soc. Psychol. 70, 491–512. doi: 10.1037/0022-3514.70.3.491

[ref25] Goffman E. (1963). Stigma: Notes on the Management of Spoiled Identity. Englewood Cliffs: Prentice-Hall.

[ref26] Goffman E. (1967). Interaction Ritual: Essays on Face-to-Face Behavior. Garden City: Anchor Books/Doubleday.

[ref27] Gurba A. N. McNair M. L. Hargreaves A. Scheerer N. E. Ng C. S. M. Lerner M. D. (2024). Editorial: break the stigma: autism. The future of research on autism stigma—towards multilevel, contextual & global understanding. Front. Psych. 15:1504429. doi: 10.3389/fpsyt.2024.1504429, 39568760 PMC11576309

[ref28] Hein P. Ansari S. (2022). From sheltered to included: the emancipation of disabled workers from benevolent marginalization. Acad. Manag. J. 65, 749–783. doi: 10.5465/amj.2020.1689

[ref29] Hennekam S. Follmer K. (2024). Neurodiversity and HRM: a practice-based review and research agenda. Equal. Divers. Incl. 43, 1119–1129. doi: 10.1108/EDI-12-2023-0424

[ref30] Hewitt J. P. Stokes R. (1975). Disclaimers. Am. Sociol. Rev. 40:1. doi: 10.2307/2094442

[ref6002] Hughes J. A. (2021). Does the heterogeneity of autism undermine the neurodiversity paradigm? Bioethics 35, 47–60. doi: 10.1111/bioe.12780 32542841

[ref31] Huws J. C. Jones R. S. P. (2010). ‘They just seem to live their lives in their own little world’: lay perceptions of autism. Disabil. Soc. 25, 331–344. doi: 10.1080/09687591003701231

[ref32] Isom Scott D. A. (2018). “Understanding white Americans’ perceptions of ‘reverse’ discrimination: an application of a new theory of status dissonance,” in Advances in Group Processes, Editors: Thye, Shane R., Lawler, Edward J. (eds). (Bingley, U.K: Emerald Publishing Limited).

[ref33] Johnson T. D. Joshi A. (2014). Disclosure on the spectrum: understanding disclosure among employees on the autism spectrum. Ind. Organ. Psychol. 7, 278–281. doi: 10.1111/iops.12149

[ref34] Johnson T. D. Joshi A. (2016). Dark clouds or silver linings? A stigma threat perspective on the implications of an autism diagnosis for workplace well-being. J. Appl. Psychol. 101, 430–449. doi: 10.1037/apl0000058 26595753

[ref35] Jones E. E. Farina A. Hastorf A. H. Markus H. Miller D. T. Scott R. A. (1984). Social Stigma: The Psychology of Marked Relationships. New York: W.H. Freeman.

[ref36] Jones K. P. King E. B. (2014). Managing concealable stigmas at work: a review and multilevel model. J. Manag. 40, 1466–1494. doi: 10.1177/0149206313515518

[ref37] Khan M. H. Grabarski M. K. Ali M. Buckmaster S. (2022). Insights into creating and managing an inclusive neurodiverse workplace for positive outcomes: a multistaged theoretical framework. Group Org. Manag. 48, 1339–1386. doi: 10.1177/10596011221133583

[ref38] Lawrence D. G. (1976). Procedural norms and tolerance: a reassessment. Am. Polit. Sci. Rev. 70, 80–100. doi: 10.2307/1960325

[ref39] Libsack E. J. Keenan E. G. Freden C. E. Mirmina J. Iskhakov N. Krishnathasan D. . (2021). A systematic review of passing as non-autistic in autism spectrum disorder. Clin. Child. Fam. Psychol. Rev. 24, 783–812. doi: 10.1007/s10567-021-00365-1, 34505231 PMC10613328

[ref40] Longmire N. H. Vogus T. J. Colella A. (2025). Relational incongruence in neurodiverse workgroups: practices for cultivating autistic employee authenticity and belonging. Hum. Resour. Manag. 64, 59–76. doi: 10.1002/hrm.22248

[ref41] Masuch T. V. Bea M. Alm B. Deibler P. Sobanski E. (2019). Internalized stigma, anticipated discrimination and perceived public stigma in adults with ADHD. Atten. Defic. Hyperact. Disord. 11, 211–220. doi: 10.1007/s12402-018-0274-9, 30341693

[ref42] Nalavany B. A. Carawan L. W. Sauber S. (2015). Adults with dyslexia, an invisible disability: the mediational role of concealment on perceived family support and self-esteem. Br. J. Soc. Work. 45, 568–586. doi: 10.1093/bjsw/bct152

[ref43] Nario-Redmond M. R. Kemerling A. A. Silverman A. (2019). Hostile, benevolent, and ambivalent ableism: contemporary manifestations. J. Soc. Issues 75, 726–756. doi: 10.1111/josi.12337

[ref44] Odegard T. N. Dye M. (2024). The gift of dyslexia: what is the harm in it? Ann. Dyslexia 74, 143–157. doi: 10.1007/s11881-024-00308-9, 38877328

[ref45] Oliveira M. Stevanovic M. (2025). “The team members were very tolerant”: social interactional ideologies and power in an intercultural context. Multilingua 44, 421–450. doi: 10.1515/multi-2024-0108

[ref46] Ottati V. Price E. D. Wilson C. Sumaktoyo N. (2015). When self-perceptions of expertise increase closed-minded cognition: the earned dogmatism effect. J. Exp. Soc. Psychol. 61, 131–138. doi: 10.1016/j.jesp.2015.08.003

[ref47] Ovaska-Few S. (2018). Promoting neurodiversity. J. Account. 225, 46–49.

[ref48] Parcesepe A. M. Cabassa L. J. (2013). Public stigma of mental illness in the United States: a systematic literature review. Admin. Pol. Ment. Health 40, 384–399. doi: 10.1007/s10488-012-0430-z, 22833051 PMC3835659

[ref49] Patton E. (2019). Autism, attributions and accommodations: overcoming barriers and integrating a neurodiverse workforce. Pers. Rev. 48, 915–934. doi: 10.1108/PR-04-2018-0116

[ref50] Pauker K. Carpinella C. Meyers C. Young D. M. Sanchez D. T. (2018). The role of diversity exposure in whites’ reduction in race essentialism over time. Soc. Psychol. Personal. Sci. 9, 944–952. doi: 10.1177/1948550617731496

[ref51] Pettigrew T. F. Tropp L. R. (2006). A meta-analytic test of intergroup contact theory. J. Pers. Soc. Psychol. 90, 751–783. doi: 10.1037/0022-3514.90.5.751, 16737372

[ref52] Pettigrew T. F. Tropp L. R. (2008). How does intergroup contact reduce prejudice? Meta-analytic tests of three mediators. Eur. J. Soc. Psychol. 38, 922–934. doi: 10.1002/ejsp.504

[ref53] Pollock T. G. Lashley K. Rindova V. P. Han J.-H. (2019). Which of these things are not like the others? Comparing the rational, emotional, and moral aspects of reputation, status, celebrity, and stigma. Acad. Manag. Ann. 13, 444–478. doi: 10.5465/annals.2017.0086

[ref54] Quinn K. A. Mason M. F. Macrae C. N. (2009). Familiarity and person construal: individuating knowledge moderates the automaticity of category activation. Eur. J. Soc. Psychol. 39, 852–861. doi: 10.1002/ejsp.596

[ref55] Radulski E. M. (2022). Conceptualising autistic masking, camouflaging, and neurotypical privilege: towards a minority group model of neurodiversity. Hum. Dev. 66, 113–127. doi: 10.1159/000524122

[ref56] Ragins B. R. (2008). Disclosure disconnects: antecedents and consequences of disclosing invisible stigmas across life domains. Acad. Manag. Rev. 33, 194–215. doi: 10.5465/amr.2008.27752724

[ref57] Roccas S. Sagiv L. Schwartz S. H. Knafo A. (2002). The big five personality factors and personal values. Personal. Soc. Psychol. Bull. 28, 789–801. doi: 10.1177/0146167202289008

[ref6001] Romualdez A. M. Heasman B. Walker Z. Davies J. Remington A. (2021). “People Might Understand Me Better”: Diagnostic Disclosure Experiences of Autistic Individuals in the Workplace. Autism in Adulthood, 3, 157–167. doi: 10.1089/aut.2020.0063

[ref58] Sadler M. S. Meagor E. L. Kaye K. E. (2012). Stereotypes of mental disorders differ in competence and warmth. Soc. Sci. Med. 74, 915–922. doi: 10.1016/j.socscimed.2011.12.019, 22321391

[ref59] Santuzzi A. M. Keating R. T. (2022). “Neurodiversity and the disclosure dilemma,” in Neurodiversity in the Workplace, Bruyère, S. M., Colella, A. (Eds.) (New York: Routledge), 124–148.

[ref60] Santuzzi A. M. Keating R. T. Martinez J. J. Finkelstein L. M. Rupp D. E. Strah N. (2019). Identity management strategies for workers with concealable disabilities: antecedents and consequences. J. Soc. Issues 75, 847–880. doi: 10.1111/josi.12320

[ref61] Santuzzi A. M. Waltz P. R. Finkelstein L. M. Rupp D. E. (2014). Invisible disabilities: unique challenges for employees and organizations. Ind. Organ. Psychol. 7, 204–219. doi: 10.1111/iops.12134

[ref62] Shapiro J. R. Neuberg S. L. (2008). When do the stigmatized stigmatize? The ironic effects of being accountable to (perceived) majority group prejudice-expression norms. J. Pers. Soc. Psychol. 95, 877–898. doi: 10.1037/a0011617, 18808265

[ref63] Smith E. R. Miller D. A. Maitner A. T. Crump S. A. Garcia-Marques T. Mackie D. M. (2006). Familiarity can increase stereotyping. J. Exp. Soc. Psychol. 42, 471–478. doi: 10.1016/j.jesp.2005.07.002

[ref64] Solomon P. Draine J. (1995). Adaptive coping among family members of persons with serious mental illness. Psychiatr. Serv. 46, 1156–1160. doi: 10.1176/ps.46.11.1156, 8564505

[ref65] Stangor C. Schaller M. (2000). Stereotypes as Individual and Collective Representations. Stereotypes and Prejudice: Essential Readings. New York: Psychology Press, 64–82.

[ref66] Steele C. W. J. (2021). When things get odd: exploring the interactional choreography of taken-for-Grantedness. Acad. Manag. Rev. 46, 341–361. doi: 10.5465/amr.2017.0392

[ref67] Steiger S. Sowislo J. F. Moeller J. Lieb R. Lang U. E. Huber C. G. (2022). Personality, self-esteem, familiarity, and mental health stigmatization: a cross-sectional vignette-based study. Sci. Rep. 12:10347. doi: 10.1038/s41598-022-14017-z, 35725744 PMC9209478

[ref68] Szulc J. M. Staniszewska Z. (2025). The unmapped paths: a strength-based inquiry into sustainable neurodivergent careers. Career Dev. Int. 30, 714–728. doi: 10.1108/CDI-03-2025-0153

[ref69] Tadmor C. T. Hong Y. Chao M. M. Cohen A. (2018). The tolerance benefits of multicultural experiences depend on the perception of available mental resources. J. Pers. Soc. Psychol. 115, 398–426. doi: 10.1037/pspa0000125, 30035567

[ref70] Tomczak M. T. Sienkiewicz Ł. Stankiewicz K. Banasik P. (2025). The conceptual framework of the neurodiversity organisational maturity model (NOMM) to support the employability of neurodivergent individuals. Empl. Relat. 47, 660–677. doi: 10.1108/ER-03-2024-0161

[ref71] Turnock A. Langley K. Jones C. R. G. (2022). Understanding stigma in autism: a narrative review and theoretical model. Autism Adulthood 4, 76–91. doi: 10.1089/aut.2021.0005, 36605561 PMC8992913

[ref72] van Knippenberg A. Dijksterhuis A. (2000). Social categorization and stereotyping: a functional perspective. Eur. Rev. Soc. Psychol. 11, 105–144. doi: 10.1080/14792772043000013

[ref73] Volpone S. D. Avery D. R. Wayne J. H. (2022). “Shaping organizational climates to develop and leverage workforce neurodiversity,” in Neurodiversity in the Workplace, Bruyère, S. M., Colella, A. (Eds.) (New York: Routledge), 16–59.

[ref74] Washburn E. K. Joshi R. M. Binks-Cantrell E. S. (2011). Teacher knowledge of basic language concepts and dyslexia. Dyslexia 17, 165–183. doi: 10.1002/dys.426, 21290479

[ref75] Wissell S. Karimi L. Serry T. Furlong L. Hudson J. (2022). “You Don’t look dyslexic”: using the job demands—resource model of burnout to explore employment experiences of Australian adults with dyslexia. Int. J. Environ. Res. Public Health 19:10719. doi: 10.3390/ijerph191710719, 36078435 PMC9518213

[ref76] Zhang R. Wang M. S. Toubiana M. Greenwood R. (2021). Stigma beyond levels: advancing research on stigmatization. Acad. Manag. Ann. 15, 188–222. doi: 10.5465/annals.2019.0031

